# Evidence that flocking behavior is rewarded by singing, flock mates, and mu opioid receptors in the nucleus accumbens

**DOI:** 10.1371/journal.pone.0318340

**Published:** 2025-01-28

**Authors:** Alyse N. Maksimoski, Taviah A. Levenson, Changjiu Zhao, Lauren V. Riters

**Affiliations:** Department of Integrative Biology, University of Wisconsin-Madison, Madison, Wisconsin, United States of America; Claremont Colleges, UNITED STATES OF AMERICA

## Abstract

It has been proposed that social groups are maintained both by reward resulting from positive social interactions and by the reduction of a negative state that would otherwise be caused by social separation. European starlings, *Sturnus vulgaris*, develop strong conditioned place preferences for places associated with the production of song in flocks outside the breeding season (gregarious song) and singers are motivated to rejoin the flock following removal. This indicates that the act of singing in flocks is associated with a positive affective state and raises the possibility that reward induced by song in flocks may play a role in flock maintenance. The goal of this study was to begin to test this hypothesis. We found that birds that sang full songs developed stronger conditioned place preferences than non-singing birds for places associated with flock mates, indicating that singers find the presence of flock mates to be rewarding. Regardless of song rate, the presence of flock mates also induced analgesia (a reflection of the reduction of a negative state). This form of analgesia has been shown to be an indirect measure of opioid release, suggesting that the presence of flock mates may induce opioid-mediated reward. Consistent with this possibility, the numbers of mu opioid receptor immunolabeled cells in the nucleus accumbens correlated positively with measurements of gregarious song and other social behaviors. Results suggest that both gregarious song and social contact promote flock cohesion and that opioids released onto mu opioid receptors in the nucleus accumbens may play an important role.

## Introduction

Social groups are vital for survival in many species. The presence of conspecifics provides protection from predators, increases foraging efficiency, and provides opportunities for social learning [1–5]. In social species, it is proposed that social groups are maintained both by a positive affective state caused by rewarding interactions with conspecifics and by the reduction of a negative affective state that would otherwise be caused by separation from the group [6, 7]. However, how the brain reinforces sociality has only begun to be explored.

Songbirds have proven to be highly tractable for studies of sociality [7, 8]. Studies on European starlings, *Sturnus vulgaris*, suggest a close association between an individual’s affective state and flocking behavior. Outside the breeding season, starlings become highly gregarious and form large mixed-sex flocks [9]. While in these non-breeding flocks, males and females sing at high rates with song rate naturally varying across individuals. Multiple studies demonstrate that this type of song, which we call *gregarious song*, is accompanied by a positive affective state. That is, high singing birds develop strong conditioned place preferences (CPPs) for places associated with their own singing behavior, leading to the proposal that the presence of flock mates and absence of predators leads to a positive affective state that facilitates singing behavior and that the act of singing itself may be self-reinforcing [10–15]. Gregarious song production is also associated with an increased motivation to rejoin a flock following removal [16]. It is possible that the positive state linked to singing becomes associated with the flock, thereby reinforcing flocking behavior and enhancing the motivation to flock.

It is also possible that the reduction of a negative state that would otherwise be induced by isolation in this social species may reinforce flocking. In social animals, social contact has a “buffering” effect against aversive stimuli [17–19]. For example, socially housed rats emit fewer distress vocalizations when experiencing mild foot shocks than their isolated counterparts [20]. This finding also highlights that social interaction can reduce a negative state through the induction of analgesia [21–24]. Consistent with these findings, in starlings, gregarious song production correlates positively with a measure of analgesia (the latency to retract a foot from hot water) [14], raising the possibility that singing in flocks may reinforce flocking by reducing a negative state.

Opioid neuromodulators may play a role in both the induction of a positive and reduction of a negative state associated with flocking. Opioids that bind to mu opioid receptors (MORs) are well known to both induce positive states and reduce negative states (e.g., by inducing analgesia). In starling flocks, peripheral administration of the MOR agonist fentanyl robustly stimulates gregarious song in males and females [10]. Moreover, the type of analgesia that correlates with gregarious song is opioid-mediated [14], suggesting that singing in flocks releases endogenous opioids and that these opioids may reinforce flocking by both the induction of a positive and the reduction of a negative state. One site in which opioids act on MORs to do this is the nucleus accumbens (NAc) [25–29]. In starlings as in mammals, infusion of a MOR agonist in the NAc results in reward as reflected in the development of a CPP [30]. Stimulation of MORs in the NAc in male and female starlings also stimulates gregarious singing behavior and promotes behaviors used to maintain social spacing (e.g., displacements) [30, 31].

Together, the results of several studies suggest that flocking in starlings may be reinforced by the induction of a positive state and/or the reduction of a negative state, and that this might involve MORs in the NAc. To test this hypothesis, we first used a CPP test to measure reward induced by the presence of flock mates and the degree to which this differs in non-singers and singers. We then explored the degree to which the presence of flock mates induces opioid-mediated analgesia (i.e., reduces a negative state). Finally, we measured the degree to which immunolabeling for MOR in NAc correlated with song and other behaviors observed in flocks.

## Methods

### Animal housing

All animal procedures were performed following protocols approved by the University of Wisconsin-Madison Institutional Animal Care and Use Committee (Protocol: L006596) and adhered to National Institutes of Health guidelines.

Adult starlings were trapped on a local farm in Madison, WI in winter 2021 and housed indoors in cages containing up to 5 same-sex birds. Prior to the start of the experiment, birds were individually color banded for identification and housed on photoperiods of 18h light:6h dark to induce photorefractoriness [32], a nonbreeding state in early fall in which male and female starlings begin to form flocks and sing at high rates. After at least 6 weeks of 18h light exposure, starlings were housed in indoor aviaries (2.13m × 2.4m × 1.98m) in mixed-sex flocks of 8 birds (2 experimental birds, 6 flock mates). Aviaries contained branches for perches, food, water, and bath water *ad libitum*. The experiment began after an observer behind a one-way mirror in an observation booth observed more than half (≥5 birds) of the birds in an aviary producing full song bouts (≥2 seconds) on more than two consecutive days. Flock mates (n = 8; 7 males [with testes], 1 female [with ovaries]) were used to maintain social stimulus for the experimental birds (n = 12; 5 males, 7 females), all of which were replaced as used.

### Baseline behavioral observations

A single observer collected behaviors for each experimental bird using a focal animal sampling method. Each bird was observed for 20 min once per day over five days to establish baseline behavioral tendencies. Behaviors were recorded via continuous sampling and the occurrence of each behavior was summed across all observation days for each individual. The behaviors recorded were: *song fragments* (songs lasting <2 secs), *full songs* (songs lasting ≥2 secs), *prrp calls* (which are common when a bird flies to a new location), *chatter calls* (which are common during mild agonistic interactions), *feed*, *drink*, *fly* (flights from one perch to another), *preen*, *stretch*, *beak wipe*, and initiating and receiving *approaches* (movement to within 10 cm of a conspecific), *leaves* (departure from a conspecific after more than 2 sec in proximity), *displacements* (movement to within 10 cm of a conspecific where the conspecific leaves within 1 second), *square-offs* (elevates neck and puffs out and directs attention to conspecific), and *pecks* directed at conspecifics. Behaviors were either recorded as instantaneous (calls, beak wipes, approaches, displacements, square offs, pecks) or as a bout (sing, feed, drink, fly, preen, stretch) separated by either a change in behavior or >1 second of rest. Additionally, the total time spent singing full song bouts was recorded in seconds and summed across all days.

### Measurement of social reward

**Fig 1:**
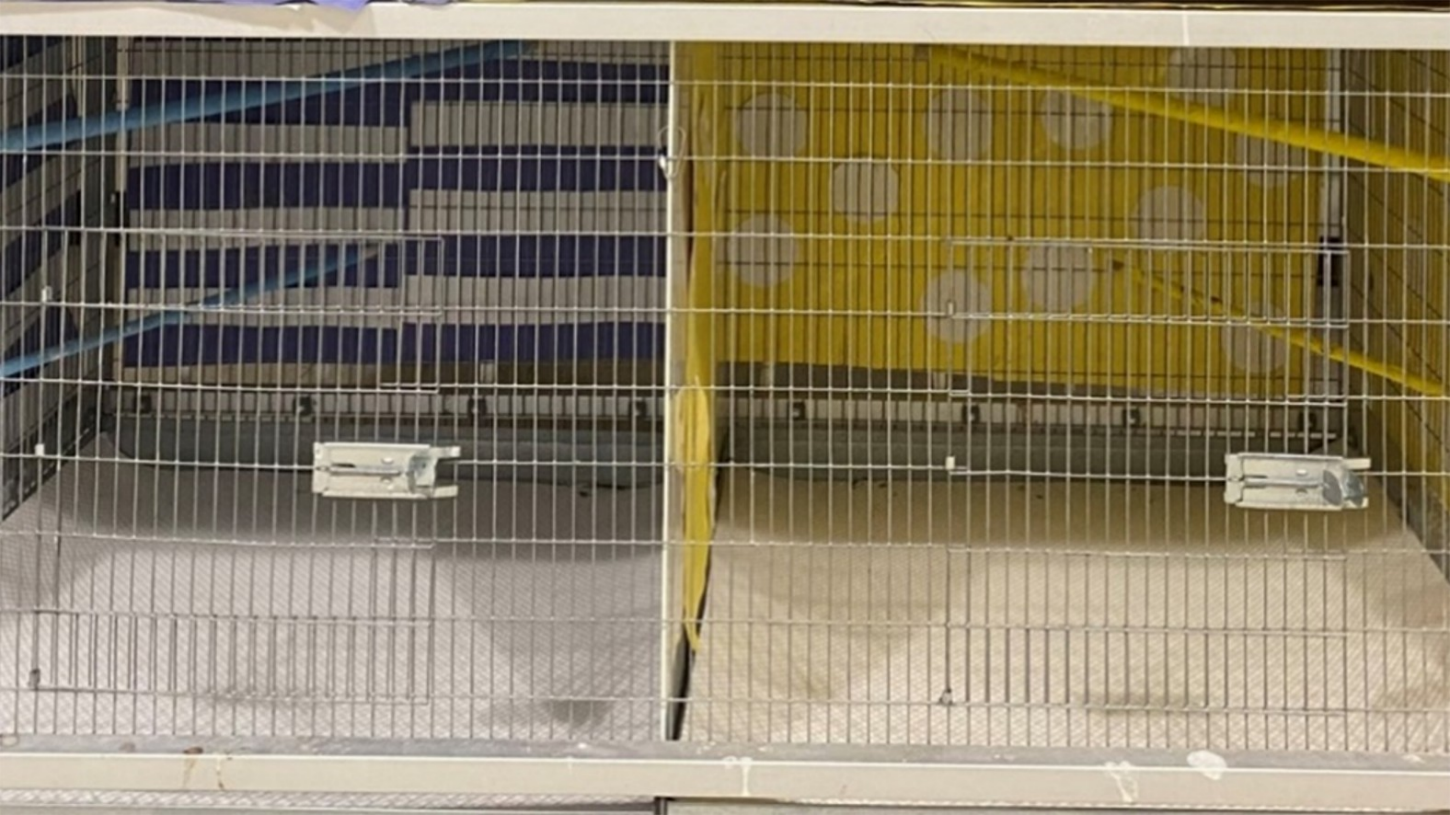
Photograph of two-chamber CPP apparatus. The CPP apparatus used consisted of two distinctly decorated conditioning chambers (e.g., blue with white stripes on the left, yellow with white dots on the right) separated by a removable partition.

Three days following the completion of the baseline behavioral observations, we used a conditioned place preference (CPP) test to assess social reward reflected in the degree to which starlings develop a preference for a place associated with flock mates compared to a place associated with separation. To do this, we used a two-chambered CPP paradigm. The CPP apparatus consisted of two distinctly decorated chambers (yellow with white dots and blue with white stripes) separated by an opaque barrier (Fig 1).

On habituation day 0, each focal bird was captured and placed singly into the center of the CPP apparatus and allowed to freely explore the yellow and blue chambers for 15 mins and then returned to its home aviary. On alternating conditioning days 1–4, a focal bird was placed into one of the distinctly decorated chambers with either two non-focal birds from its aviary (*flock-conditioned chamber*) or with no social stimuli (*separation-conditioned chamber*) for 45 minutes such that each bird was conditioned twice to each chamber. The chamber/stimulus pairings and order were counterbalanced across birds. After conditioning each day, birds were returned to their home aviaries. In this study, we had two CPP test cages and conditioned four birds at a time, where two birds were conditioned to the flock-conditioned chambers while another two birds were conditioned to the separation-conditioned chambers. The testing of four birds at the same time created a situation in which the bird in the separation-conditioned chambers could hear birds moving in the neighboring cages, but neither see nor touch the other birds.

On test day 5, focal birds were tested singly. For each test, the divider between the chambers within each CPP apparatus was removed to grant each bird unrestricted access to both empty chambers. Each bird was captured from its flock and placed into the CPP apparatus. An observer video recorded the bird for 15 mins and quantified the time spent in each of the chambers as a measure of place preference.

### Social analgesia measure

Within two weeks following the completion of CPP testing, focal birds were administered a series of opioid-mediated analgesia tests [14]. In short, each subject was held by an experimenter, hooded with a soft cloth, and had its foot quickly lowered into a heated water bath (50–55°C). The latency to withdraw the foot from the water (i.e., analgesia) was measured. The maximum time allowed for withdrawal was 30 secs, after which the foot was manually removed from the bath. After testing, the subject’s foot was dipped into room temperature water (20–25°C) for 30 secs and the subject was placed back into its aviary.

Analgesia was measured in each bird after two conditions, following an ABAB design. On the first day, a focal bird was captured from its flock, foot dipped (*flock*), and then immediately moved to a neighboring aviary that was identical to its home aviary, with the same number of perches, food, and water *ad libitum*, but with no flock mates present. The bird was housed in this aviary alone for 24 hours (±1 hour), after which, it was captured and administered the foot dip test (*separation*) before being returned to its home aviary. Birds were then housed in their home aviaries for 24 hours (±1 hour) and the process was repeated. Additionally, we also assessed analgesia 20 mins after each bird was reunited with its flock but did not see any effect so will not discuss these results further (but see S1 Table). Starlings that are visually separated from conspecifics have elevated levels of the stress hormone corticosterone [33]. Previous work has demonstrated that acute stress (such as that induced by separation) releases opioids and induces analgesia; however, longer-term stress (such as that induced by overnight separation) reduces analgesia [34, 35]. Therefore, we tested analgesia 24 hours after separation, based on Panksepp [20], which suggests that this is when separation would decrease analgesia. Additionally, pilot testing confirmed that birds experienced decreased analgesia after 24 hours compared to baseline.

### Immunolabeling for mu opioid receptors

After the completion of all behavioral tests, focal birds were returned to their flocks for at least 24 hours and then placed into an empty aviary for 24 hours. Half were then sacrificed and brains collected after one additional hour in the empty aviary (separation condition) and half were sacrificed and brains collected after being reunited with the flock for one hour. This was done to explore the effects of separation and reunion on immediate early gene activity as part of another study not discussed here.

After the separation or reunion condition, birds were deeply anesthetized with isoflurane and perfused with 4% paraformaldehyde (PFA) in 0.1 M phosphate buffer (PB; pH 7.4). Following perfusion, brains were extracted and submerged in 4% PFA overnight. The next day, tissue was transferred to a 30% sucrose solution until saturation, after which tissue was frozen at -80°C until sectioning. Tissue was sectioned at 40 μm using a Leica CM1850 cryostat (Leica Biosystems, Wetzlar, Germany) and stored in cryoprotectant antifreeze solution at -20°C until labeling.

We ran immunolabeling for mu opioid receptors (MORs) on three sections containing the most rostral portion of the NAc, which is the location for the avian NAc proposed by Reiner [36], which is a site in which we find stimulation of MOR to both induce reward and singing behavior [30, 31]. This region receives a TH positive projection from the ventral tegmental area [30] and contains dense enkephalin and tyrosine hydroxylase labeling, supporting homology with the mammalian NAc [37]. Fluorescent Immunolabeling was conducted following methods similar to those used in past studies from our lab [38, 39]. Briefly, sections were rinsed 5× for 5min in 0.02M phosphate-buffered saline (PBS) and then incubated in 1.5% H_2_O_2_ / 50% methanol for 30min to inhibit endogenous peroxidase activity. The tissue was then washed 3× for 10min with normal goat serum (NGS). Following washes, tissue was blocked for 1hr before being incubated at 4°C for two days in rabbit anti-MOR (1:5000, ab10275, Abcam) in primary antibody incubation solution (PAIS). After primary incubation, sections were washed 3× for 10min in TBST, then incubated in HRP-conjugated Goat anti-rabbit antiserum for 1hr (1:100, Cell Signaling Technology #7074) in TBST. Sections were again washed 3× for 10min in TBST and labeled by incubation for 10 min in Cy3-conjugated tyramide (TSA Plus Cyanine 3 kit; Perkin Elmer; Waltham, MA), followed by 3×10min washes in TBST. Sections were then mounted onto gel-coated slides, dehydrated, and cover-slipped.

**Fig 2:**
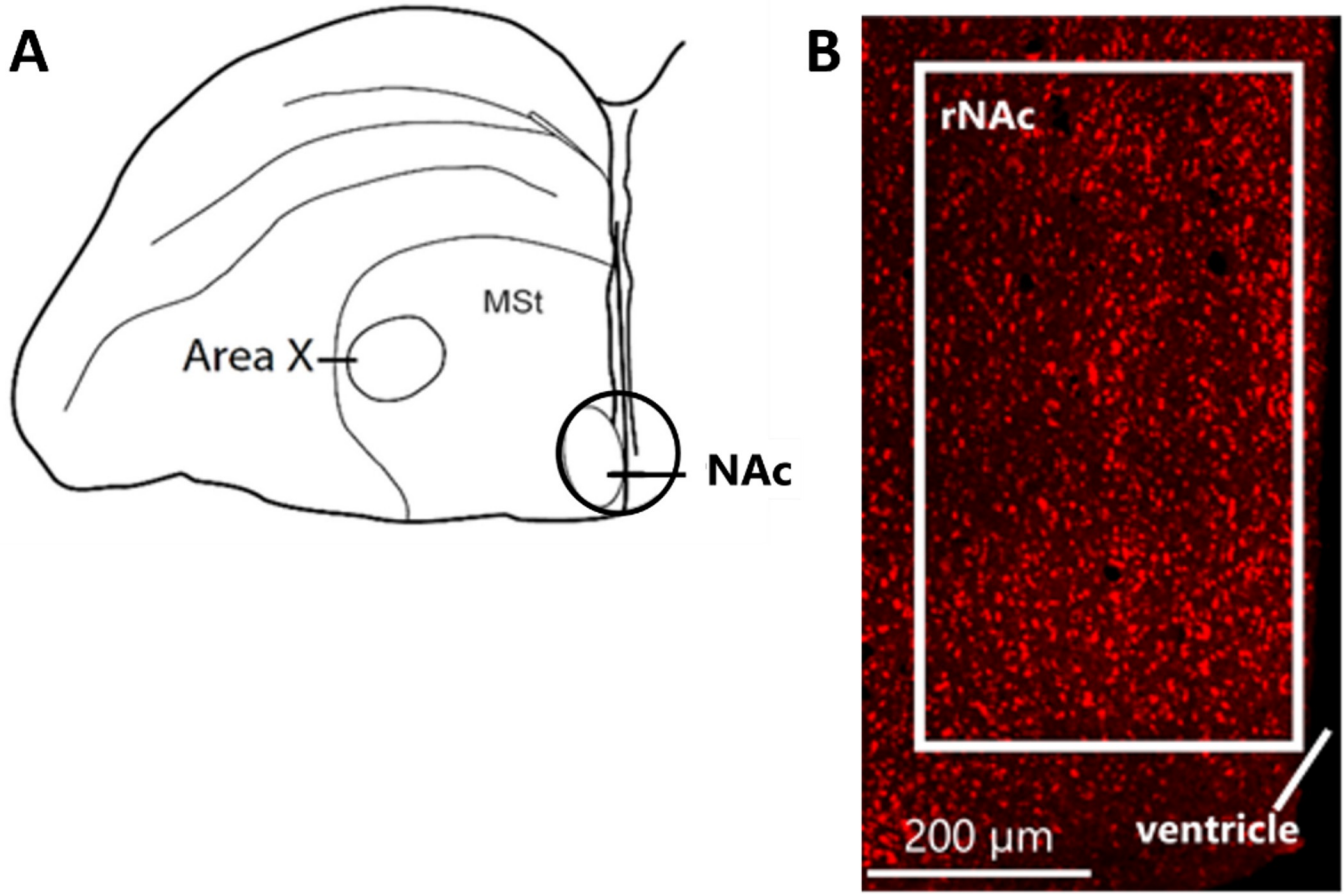
Visualization of the nucleus accumbens location used for immunolabeling. Location of the nucleus accumbens (NAc) on a coronal section (**A**) with corresponding mu opioid receptor label location on a confocal image (**B**). Abbreviations: MSt = medial striatum.

Photos of the immunolabeled sections were taken at 1024 × 1024 pixels one hemisphere at a time with a Zeiss LSM 710 Meta laser scanning confocal microscope at 40X (Zeiss; Oberkochen, Germany). All cell quantification was conducted using QuPath 0.4 [40] by an observer blind to the condition and behavior of each bird. Counting of labeled cells in the NAc was performed using a rectangle (480 x 890 μm). In short, each raw NAc image was loaded into QuPath and the uniform rectangle was overlayed into the correct position (Fig 2) within the bounds of the NAc. Automated cell detection was performed using a background rollerball radius of 3 μm to reduce noise and a minimum measure area of 10 μm^2^ to reduce false positives of non-cells. The minimum intensity of MOR stain within each counted cell was set between 150 and 300 to ensure only bright labels were counted, adjusted for each image. Counts within a single region were summed across both hemispheres and all three sections for each bird.

### Statistical analyses

All statistical analyses were conducted using GraphPad Prism 10.2 (GraphPad Prism version 10.2 for Windows, GraphPad Software, San Diego, California USA) or jamovi (2.4, jamovi project, https://www.jamovi.org) with details provided in the results section. Significance is denoted with α<0.05 for all analyses. Kolmogorov-Smirnov tests indicated that data did not violate normality. Levene’s tests indicated that homogeneity of variance was violated for some baseline behavioral measures (i.e., approach, square off, receive square off, and fly) and for time spent on the flock paired side in the CPP analysis; however, non-parametric and parametric tests revealed similar patterns of results so we used parametric analysis for consistency. Given that we expected differences related to song production (as detailed in the introduction), for analysis we separated birds into “non-singers” (n = 3) and “singers” (n = 9) based on whether they ever produced a full song bout during baseline behavior observations. There was not adequate statistical power to analyze sex because when divided into non-singers and singers, there were only 1 male and 2 females in the non-singer group. We observed no trends for sex differences (which matches our prior studies on gregarious song [10, 16, 30, 31, 41]), combined males and females for analysis, and we indicate sex in figures. Full raw data are contained within S1 Table.

## Results

### Baseline behaviors in flocks

To begin to explore behavioral differences in non-singers (n = 3) and singers (n = 9) we ran a repeated measures ANOVA (repeated to account for non-independence of the multiple behavior measurements) with non-singers/singers entered as a categorical variable and behaviors (minus full song measures [time singing and song bouts], which were what we used to divide the groups) entered as non-independent, repeated measures variables. Analysis revealed no significant main effect for non-singers/singers (F_1,10_ = 3.95, p = 0.0749) or interactions between behavior and non-singers/singers (F_18,180_ = 1.62, p = 0.060). A main effect was found for behavior (F_18,180_ = 6.38, p = 0.000), indicating that birds performed different behaviors at different rates (*Table 1*).

**Table 1:** Descriptive statistics for behavioral data, separated by non-singers (n = 3) and singers (n = 9). Behavioral data are represented in behavior rate, apart from time spent singing which is represented in seconds. Asterisk denotes statistical significance (p<0.05).

	non-singers			singers		
	[min, max]	median	mean ±SEM	[min, max]	median	mean ±SEM
**approach conspecific**	[2, 4]	4	3.3 ±0.7	[3, 28]	11	14.7 ±3.5
**receive approach**	[0, 6]	0	2.0 ±2.0	[4, 39]	11	12.8 ±3.5
**leave**	[2, 6]	2	3.3 ±1.3	[3, 24]	13	12.9 ±2.4
**receive leave**	[0, 2]	2	1.3 ±0.7	[1, 31]	12	12.6 ±3.2
**displace conspecific**	[2, 8]	2	4.0 ±2.0	[2, 31]	7	10.9 ±3.3
**peck conspecific**	[1, 2]	1	1.3 ±0.3	[0, 29]	5	7.9 ±3.0
**square-off**	[0, 1]	1	0.7 ±0.3	[0, 8]	3	3.0 ±1.0
**receive displacement**	[3, 11]	7	7.0 ±2.3	[7, 26]	12	14.0 ±2.1
**receive peck**	[0, 1]	0	0.3 ±0.3	[0, 13]	3	4.2 ±1.4
**receive square-off**	[0, 1]	0	0.3 ±0.3	[0, 10]	2	3.1 ±1.2
**song fragment**	[0, 8]	8	5.3 ±2.7	[7, 83]	15	31.8 ±10.0
**song bouts**	[0, 0]	0	0 ±0	[1, 56]	6	19.0 ±7.2
time singing*	[0, 0]	0	0 ±0	[4, 1358]	102	433 ±171
**prrp call**	[1, 11]	1	4.3 ±3.3	[0, 61]	23	28.8 ±7.3
**chatter call**	[0, 5]	1	2.0 ±1.5	[0, 18]	3	5.3 ±2.0
**feed**	[15, 43]	31	29.7 ±8.1	[17, 84]	23	29.3 ±7.1
**drink**	[11, 15]	15	13.7 ±1.3	[0, 15]	8	7.6 ±1.5
**beak wipe**	[36, 62]	52	51 ±7.9	[14, 61]	46	42.9 ±4.8
**fly**	[37, 41]	37	38.3 ±1.3	[24, 345]	70	138 ±41.2
**preen**	[26, 79]	28	44.3 ±17.3	[39, 91]	55	64 ±7.0
**stretch**	[3, 11]	4	6.0 ±2.5	[3, 15]	6	8.1 ±1.4

### Social reward

**Fig 3:**
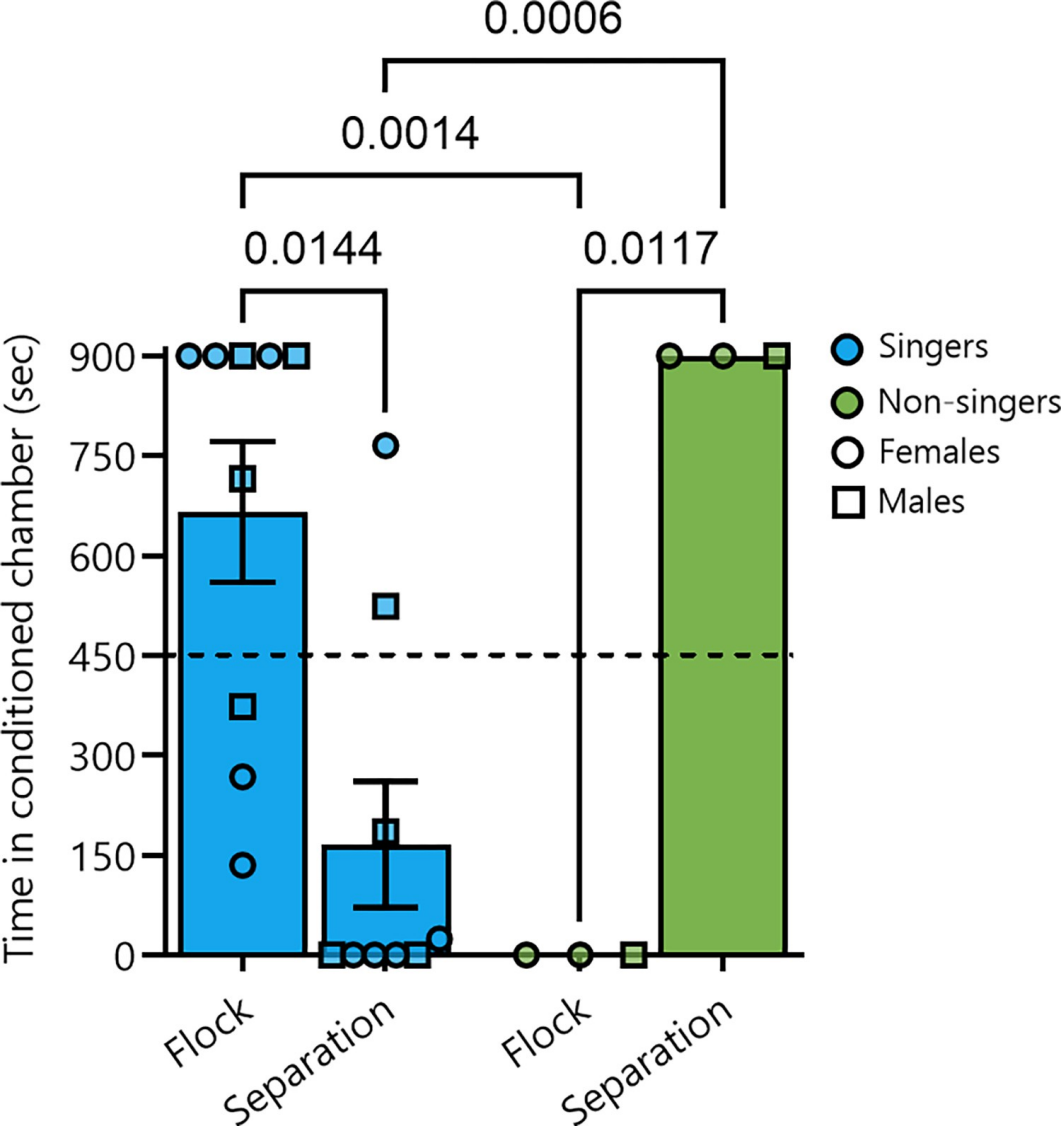
Singers developed a strong place preference for a place associated with flock mates, and non-singers preferred separation. Starlings that sang full songs (singers, left) spent more time on the flock-conditioned side of the CPP apparatus compared to the separation-conditioned side. Birds that did not sing full song bouts (non-singers, right) spent more time on the separation-conditioned side than the flock-conditioned side. The Y axis shows the time spent in each chamber (flock, separation) in seconds, with the dotted horizontal line at 450 seconds denoting equal time in each chamber. The X axis shows the conditioned chamber (flock, separation) for each group (singers, non-singers). Numbers denote significant p-values (p<0.05). Singers are shown in blue, non-singers in green, females by circles, and males by squares.

A repeated measures ANOVA with time spent on the conditioned side of the CPP apparatus (flock, separation) entered as a non-independent, repeated measures variable and song level (non-singers, singers) entered as a categorical variable revealed a significant conditioned side x song level interaction (F_1,10_ = 17.16, p = 0.0020) but no significant main effects for conditioned side (F_1,10_ = 1.41, p = 0.2628) or song level (F_1,10_ = 0.32, p = 0.5864). Post-hoc Fishers LSD tests revealed that singers spend significantly more time on the flock-conditioned side than the separation-conditioned side (p = 0.0144), and more time on the flock-conditioned side than non-singers (p = 0.0014). Non-singers spent significantly more time on the separation-conditioned side than the flock-conditioned side (p = 0.0117), and more time on the separation-conditioned side than singers (p = 0.0006; Fig 3).

### Social analgesia

**Fig 4:**
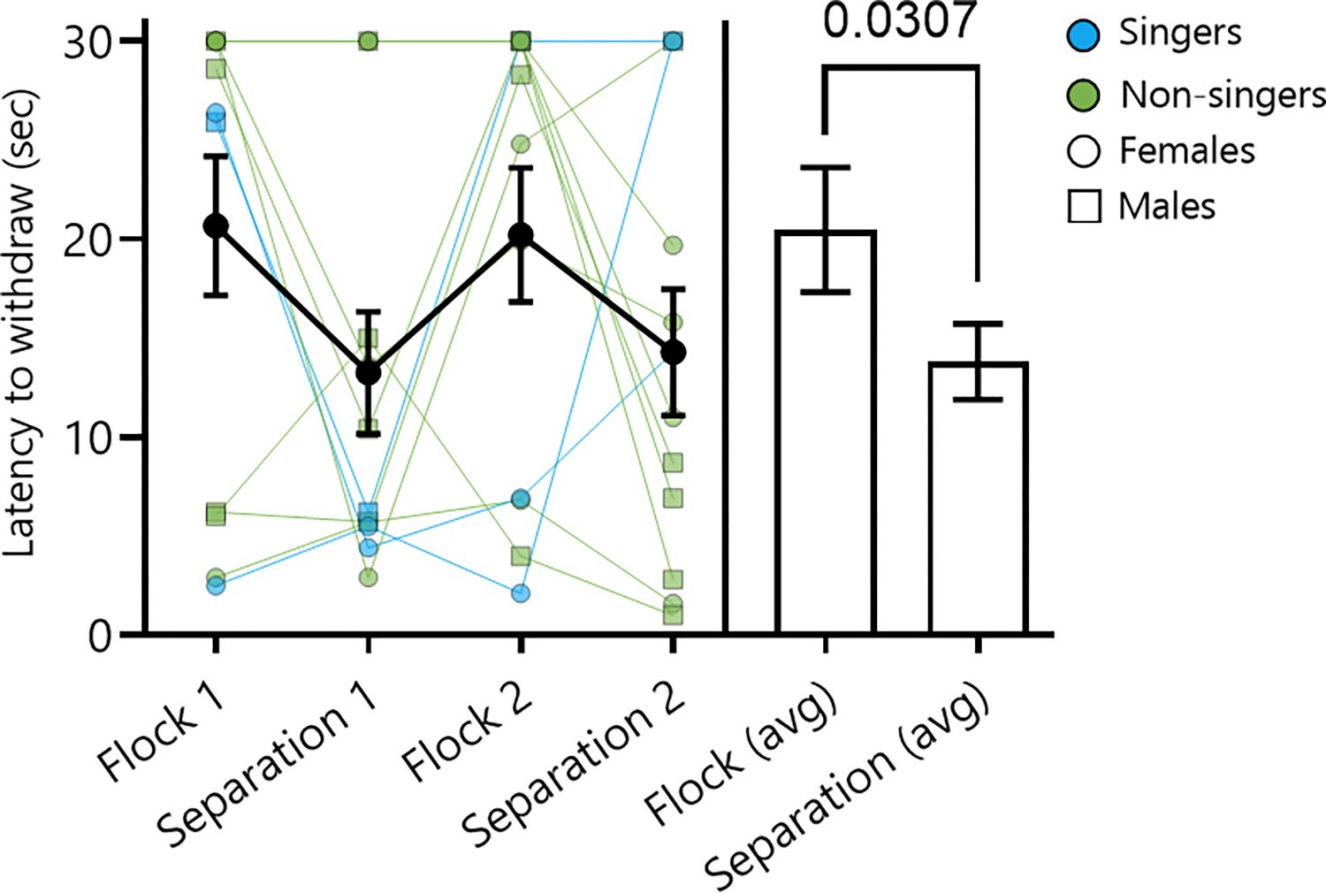
Flocking induces analgesia. Starlings removed their foot from hot water more quickly after being separated from the flock overnight (separation), compared to when they were housed in the stable flock (flock). The Y axis shows the latency to withdraw foot from a hot water bath. The X axis shows repeated tests in flock (1, 2) and separation (1, 2) conditions following an ABAB design, as well as the mean from the flock and separation conditions. Analysis was performed on the mean of each test (flock avg, separation avg; see text for details). Numbers denote significant p-values (p<0.05). Thick black lines show the mean latency to withdraw across all birds for each measurement and error bars represent the SEM. Singers are shown in blue, non-singers in green, females by circles, and males by squares.

A repeated measures ANOVA was run with each of the two analgesia tests (i.e., latency to retract a foot from hot water) for birds in each condition (separation test 1, 2; flock test 1, 2) entered as repeated measures variables and song level (non-singers, singers) entered as a categorical variable. There were no main effects for song level (F_1,10_ = 0.20, p = 0.6661), no main effects for test 1 compared to test 2 for the flock condition (F_1,10_ = 2.39, p = 0.1528), and no main effects for Test 1 compared to Test 2 for the separation condition (F_1,10_ = 0.73, p = 0.4123). Therefore, we pooled non-singers and singers and for each individual compared the mean of the two analgesia responses for each condition. A repeated measures paired t-test revealed that analgesia was higher (i.e., the latency to retract a foot from hot water was longer) in birds from stable flocks than after separation (t_11_ = 2.48, p = 0.0307; flock condition mean ±SEM = 20.47 ±3.15 secs; Separation condition mean ±SEM = 13.80 ±1.91 secs; Fig 4).

### Relationships between mu opioid receptor labeling and behaviors

We used Principal Component Analysis (PCA) with varimax rotation to reduce the multiple behavioral variables to a smaller set. Analysis identified six Principal Components (PCs) with eigenvalues >1.0, accounting for a cumulative variance of 93.0% (*Table 2*). PC1 had high loadings (>0.60) for behaviors associated with dominance (peck, square-off, receive approach, receive leave, and chatter call), therefore we call this PC *Agonistic Behavior*. PC2 had the highest loading for displacements and high loadings for other behaviors associated with dynamic social movement within the flock (fly, leave, approach, and prrp call), therefore we call this PC *Social Spacing* (as in [31]). PC3 had high loading values for stretch, preen, and beak wipes, therefore we call this PC *Grooming*. PC4 had high loading values for receiving agonistic behaviors from others (receiving displacements and pecks), therefore we call this PC *Submission*. PC5 had high loading values for number of song bouts and time spent singing, so we call this PC *Song*. PC6 contained high loading values for feed and drink, so we call this PC *Feed/Drink* (*Table 3*).

**Table 2:** Principal component analysis eigengvalues and variance for each component.

Component	Eigenvalue	% Variance	Cumulative Variance
**1**	8.36	41.8	41.8%
**2**	3.54	17.7	59.5%
**3**	2.31	11.5	71%
**4**	2.18	10.9	81.9%
**5**	1.18	5.9	87.8%
**6**	1.04	5.2	93%

**Table 3:** Principal component analysis results with the loadings >0.60 for each selected behavior in bold.

	PC1: Agonistic Behavior	PC2: Social Spacing	PC3: Grooming	PC4: Submission	PC5: Song	PC6: Feed/Drink
**peck conspecific**	**0.9447**	-0.0259	0.1114	0.0842	-0.0173	0.00757
**receive square-off**	**0.9325**	0.2234	-0.1143	0.07556	-0.1336	-0.1394
**receive approach**	**0.9059**	0.0494	0.1041	0.31534	0.01414	-0.0729
**square-off**	**0.8683**	0.3483	-0.1731	0.06977	-0.0832	-0.1976
**receive leave**	**0.835**	0.3896	0.1233	0.36303	0.03844	0.01575
**chatter call**	**0.7206**	0.0596	0.2419	0.0868	0.4085	-0.3587
**fly**	0.0951	**0.9318**	-0.0184	0.08479	0.15258	-0.1047
**displace conspecific**	0.1803	**0.900**	0.0216	-0.20916	0.28044	-0.0323
**approach conspecific**	0.1328	**0.8928**	0.1643	0.35713	-0.0069	0.15058
**leave other**	0.1974	**0.8394**	0.0474	0.46327	-0.0037	-0.0108
**prrp call**	0.4479	**0.7125**	0.1072	0.15598	0.46749	-0.1584
**stretch**	-0.0545	-0.0237	**0.9222**	0.18802	-0.144	0.0989
**preen**	0.3923	0.0208	**0.8762**	-0.09532	0.03552	-0.1441
**beak wipe**	-0.2334	0.3243	**0.6009**	-0.39625	0.20976	-0.0229
**receive displacement**	0.4235	0.2612	0.0009	**0.84487**	0.0598	0.07958
**receive peck**	0.3751	0.4443	-0.0385	**0.67644**	0.06347	-0.3217
**song bouts**	-0.0975	0.1263	0.038	-0.00417	**0.93067**	-0.0868
**time singing**	0.014	0.5758	-0.1976	0.06143	**0.77592**	-0.0149
**drink**	-0.1376	-0.1032	-0.2149	-0.2222	0.06642	**0.84239**
**feed**	-0.1743	0.0763	0.2587	0.2395	-0.2727	**0.825**

**Fig 5:**
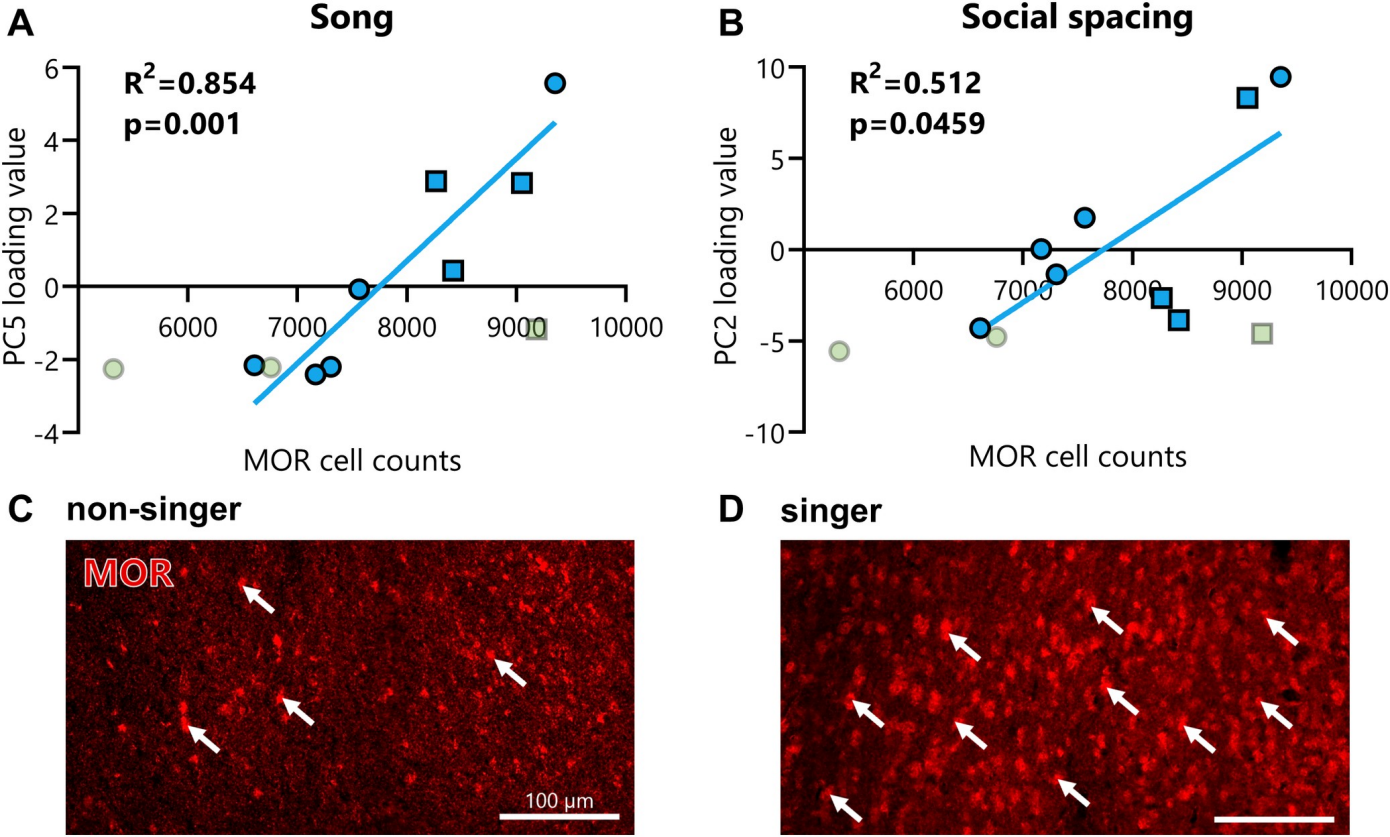
Song and social spacing principal components positively correlated with the number of MOR-positive cells in the NAc for singers. Starlings that sang full songs (singers) had significant positive correlations between the number of mu opioid receptor labeled cells in the NAc and principal components (PCs) for both Song (**A**) and Social Spacing (**B**) behaviors. The Y axes show the loading value for each PC. The X axis shows the number of MOR-positive cells counted within the bounds of the NAc. Singers are shown in blue, non-singers in green, females by circles, and males by squares. Differences in MOR immunolabeled cells are shown in representative photomicrographs of the NAc for a non-singer (**C**) and singer (**D**). Images were taken using a confocal (Zeiss 710). Arrows indicate example counts of MOR-positive cells.

Given that only singers exhibited social reward in the CPP test above, we anticipated that if MORs in the NAc underlie the reward associated with flocking, MORs in the NAc may only relate to behavior in singers. We thus ran analyses with and without non-singers. One outlier was dropped from analysis (MOR = 5372, PC2 = 7.46, PC5 = 0.742). A significant positive correlation was found between the numbers of MOR-positive cells in the NAc and PC5 Song (R^2^ = 0.544, p = 0.0096) with a trend for PC2 Social Spacing (R^2^ = 0.346, p = 0.0568) when singers and non-singers were combined. Removal of the non-singers revealed a significant positive correlation for singers between the numbers of MOR-positive cells in the NAc and both PC5 Song (R^2^ = 0.854, p = 0.001) and PC2 Social Spacing (R^2^ = 0.512, p = 0.0459; Fig 5). Here we report only the results of the singers. When sequential Bonferroni corrections were run to reduce Type I error for the analyses that include only singers, Social Spacing did not reach statistical significance. Correlations between MOR-positive cell and the other PCs were not significant with (PC1 R^2^ = 0.083, p = 0.391; PC3 R^2^ = 0.193, p = 0.177; PC4 R^2^ = 0.035, p = 0.582; PC6 R^2^ = 0.260, p = 0.109) or without non-singers (PC1 R^2^ = 0.032, p = 0.670; PC3 R^2^ = 0.001, p = 0.930; PC4 R^2^ = 0.031, p = 0.676; PC6 R^2^ = 0.116, p = 0.408).

## Discussion

Results of this study support a role for reward induced by a positive affective state, the reduction of a negative state, or both, in the reinforcement of flocking behavior. Correlational data also support and expand upon past studies that demonstrate an important role for MOR activation in the NAc in gregarious song as well as social spacing behaviors [31]. Collectively the results of the present study suggest that opioid release in the NAc triggered by the presence of flock mates and/or the act of singing may function to reinforce flocking behavior.

### Social reward differs between non-singers and singers

As in prior studies, starlings displayed strong individual differences in gregarious singing within flocks. We found that only birds that sang full bouts of gregarious song in flocks during baseline behavioral observations developed preferences for a place that had been paired with the presence of flock mates. This suggests that high, but not low, singers find the presence of flock mates to be rewarding, or that they find separation from flock mates to be aversive. These findings expand upon the results of a prior study that showed that birds that sang full bouts are also more motivated to rejoin a flock when separated, spending more time near a flock than a pair in a choice test [16]. In addition, multiple prior studies show that singers develop strong preferences for places associated with the act of producing gregarious song [10–15]. Together these findings suggest that song production is stimulated and/or maintained by a reward state, and it is possible that the reward associated with gregarious song is naturally linked to the presence of flock mates, thereby reinforcing flocking behavior.

In contrast to singing birds, the 3 non-singers in this study all developed a preference for the separation rather than the social chambers of the CPP apparatus. This suggests that either these birds find separation from flock mates to be a positive experience or that they find the presence of flock mates to be aversive. Although in future studies, the use of a 3-chamber CPP apparatus will help us to parse this out, these findings demonstrate strong differences in the affective state experienced in flocks by non-singers and singers. It is possible that past experiences in non-singers within the flock may have reduced the reward value of flock mates in these birds. For example, if they received more agonistic responses from flock mates this may have reduced social reward. This possibility is supported by the results of a past study in which starlings that received more agonistic behavior in a flock were found to be less motivated to flock than birds that received low levels of agonistic behavior [16]. Although, in the present study the 3 non-singers were not observed to receive more agonistic behavior than singing birds (in fact the trend was in the opposite direction), it is possible that negative social experiences that shape social responses may be long lasting and have occurred prior to our study. It is also possible that there are constitutive differences in non-singers and singers that underlie differences in social motivation and reward that are not a consequence of prior experience. Perhaps age plays a role in the production of song and/or social reward, however as these are wild caught birds, there is no reliable way to determine age prior to the inclusion in this study. This is a potential avenue for future research.

### Flocking is associated with opioid-mediated analgesia in both non-singers and singers

Starlings demonstrated elevated analgesia (i.e., maintained a foot in hot water longer) when in flocks compared to after being separated from flock mates for 24 hours. Prior studies demonstrate this measure of analgesia to be opioid-sensitive [14, 42]. Thus, the present results suggest that for both non-singers and singers the presence of flock mates may induce reward through opioid release and the reduction of a negative state (i.e., the induction of analgesia). Previous research using the same measure of analgesia showed that gregarious song production correlated positively with analgesia [14]; however, birds in the present study did not sing prior to the analgesia test. This suggests that the present results are independent of singing behavior. The finding that, unlike the social CPP, social analgesia did not differ by song level singers suggests that flocking may be reinforced by different mechanisms in non-singers and singers. One possibility is that non-singers may primarily remain in flocks to avoid a negative affective state, whereas, for singers flocking may be dually reinforced by both the reduction of negative state and by the induction of a positive state by the presence of flock mates. It may also be that results reflect methodological differences. Birds were only separated from conspecifics for 45 minutes during the CPP conditioning but for 24 hours for analgesia testing. Perhaps the increased time away from the flock surpassed the lack of separation aversion for non-singers. These possibilities should be explored in future studies.

### MOR immunolabeling in NAc correlated with gregarious song and social spacing

In birds that sang full songs, the number of MOR-positive cells in the NAc correlated positively with a principal component with high loadings for gregarious song and a component with high loadings for behaviors associated with social spacing (i.e., displacements, leaves, and approaches). These findings suggest a possible causal role for MORs in the NAc in these behaviors that is supported by previous studies in which intra-NAc administration of a MOR agonist stimulated both gregarious song production and behaviors used to maintain social spacing in flocking starlings [30, 31]. Past studies also found that stimulation of MORs in the NAc induced a CPP in starlings and rodents [28, 30, 43] and in rodents has been found to induce analgesia [25, 44] and reduce stress-related behaviors [45]. Together these findings are consistent with a role for MORs in the NAc in gregarious song, social spacing, and reinforcement that may promote and maintain flocking behavior. In this study, we focused on a rostral portion of the NAc [36] that was the site in which MOR stimulation in starlings stimulated gregarious song and CPP (detailed in methods). Future studies are now needed to explore roles for regions considered homologues of the mammalian accumbens core and shell.

## Conclusions

It has been proposed in gregarious species that groups may be dually maintained by a positive affective state caused by interactions with conspecifics as well as the reduction of a negative affective state that would otherwise be caused by separation from the group. The present study offers support for this hypothesis and uncovers a potential key role for MORs in the NAc.

## Supporting information

S1 Table Latency to withdraw including reunion. Descriptive statistics of withdrawal times (in seconds) for all birds separated by song level, with the addition of the reunion measurement taken twenty minutes after reintroduction to the home flock.
(XLSX)

S2 Table Minimal data set. Raw data for each of twelve subjects, including song level (binary), behaviors (counts), CPP test times (seconds), withdrawal times (seconds), and MOR cells (counts). Behavioral data are represented in behavior rate, apart from time spent singing which is represented in seconds. MOR cell counts are represented in total counts across all stained sections.
(XLSX)
